# Radiography Interns' and Recent Graduates' Self‐Perceived Professional Competency Development at Two Saudi Universities: A Cross‐Sectional Study

**DOI:** 10.1002/jmrs.70118

**Published:** 2026-09-22

**Authors:** Ahmad Subahi, Khalid Mahboob, Refan Dammas, Wejdan Aljehani, Majid Al Thaqafy, Khalid Alshamrani, Ahmaed Baashar

**Affiliations:** ^1^ Basic Science Department, College of Science and Health Professions King Saud Bin Abdulaziz University for Health Sciences Jeddah Saudi Arabia; ^2^ King Abdullah International Medical Research Center Jeddah Saudi Arabia; ^3^ Ministry of National Guard—Health Affairs Jeddah Saudi Arabia; ^4^ Department of Basic Sciences, College of Science and Health Professions King Saud Bin Abdulaziz University for Health Sciences Jeddah Saudi Arabia; ^5^ College of Applied Medical Sciences King Saud Bin Abdulaziz University for Health Sciences Jeddah Saudi Arabia; ^6^ College of Medicine King Saud Bin Abdulaziz University for Health Sciences Jeddah Saudi Arabia

**Keywords:** interns, medical imaging, medico‐legal and ethical knowledge, patient safety, professional competencies, professional competency development, radiographers, radiography, radiological sciences, Saudi Arabia

## Abstract

**Introduction:**

Patient safety is a fundamental component of healthcare quality and is closely linked to the preparation of healthcare graduates for professional practice. In radiography undergraduate programs, students are expected to develop clinical, technical, communication, teamwork, patient care, ethical, and image interpretation‐related competencies required for safe and effective practice. This study assessed radiography interns' and recent graduates' perceptions of acquired knowledge and skills across seven professional competency domains during their undergraduate education.

**Methods:**

A cross‐sectional descriptive online survey was conducted among 61 radiography interns and recent graduates from the 2020 to 2023 cohorts at two Saudi universities. A self‐administered questionnaire assessed perceived improvement, program coverage, confidence, clinical placement contribution, perceived importance, and future relevance across seven domains: communication, procedural knowledge, technical knowledge, patient care, collaborative teamwork, medico‐legal and ethical knowledge, and identification of significant findings. Data were analysed using descriptive statistics, including frequencies, percentages and Likert‐scale mean scores.

**Results:**

Participants generally reported positive perceptions of professional competency development across most domains, particularly communication, procedural knowledge, technical knowledge, patient care and collaborative teamwork. These domains were also perceived as important and relevant to future professional practice. However, medico‐legal and ethical knowledge and identification of significant findings received comparatively lower scores in perceived improvement, program coverage and confidence.

**Conclusion:**

This study provides perception‐based insight into radiography undergraduate education at two Saudi universities. Participants reported generally positive perceptions across most professional domains, while lower scores in medico‐legal and ethical knowledge and identification of significant findings highlight opportunities for targeted curricular enhancement to support graduate preparedness.

## Graphical abstract

This cross‐sectional study explored radiography interns' and recent graduates' perceptions of professional competency development at two Saudi universities. Positive perceptions were reported across most domains, while medico‐legal and ethical knowledge and identification of significant findings were identified as areas for targeted curricular and clinical enhancement. This is to disclose that Graphical Abstract designed by ChatGPT (only design not the content).

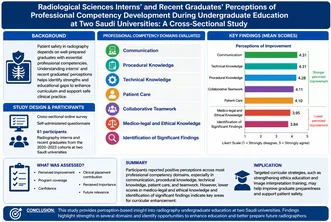

## 1 Introduction

Patient safety refers to the prevention of harm to patients and the minimization of unnecessary risks associated with healthcare [1]. It is the top priority for all healthcare providers, including physicians, radiographers, nurses and others. Ensuring patient safety is essential for maintaining public confidence in healthcare systems and for ensuring that individuals who seek medical care receive safe and high‐quality services. The impact of medical errors and adverse events on patients and healthcare providers is far‐reaching. These incidents can lead to severe consequences, including injury to the patient, increased healthcare costs, and legal issues for the healthcare provider or organization [2].

In radiography practice, patient safety is closely associated with the competence of radiographers. Radiographers are involved in clinical activities that require accurate procedural knowledge, technical knowledge, patient positioning, radiation protection, communication with patients and healthcare staff, ethical decision‐making, patient care, and identification of significant imaging findings [3, 4]. Therefore, any lack of knowledge or skills in these areas may affect the quality and safety of medical imaging practice. For example, inadequate procedural or technical knowledge may contribute to inappropriate imaging practice or unnecessary repeat imaging, while poor communication or limited ability to identify significant findings may delay appropriate clinical action. For this reason, preparing radiography graduates with the required knowledge and skills is important for supporting safe and effective healthcare practice [1, 3, 5].

Recently, there have been many concerns regarding the quality of education received by students and healthcare workers, as well as the perceived preparedness of recent graduates and interns.

A study has shown that a significant percentage of medical errors can be attributed to trainees, with 78% of fourth‐year students and 98% of residents frequently blaming medical errors [6]. This underscores the importance of understanding what recent graduates and interns think about the knowledge and skills they have acquired. By gathering information about students' perceived knowledge, skills, and educational needs, educators can develop improved teaching methods, strengthen graduate preparedness, and support safer clinical practice [5].

This study is informed by the concept of students' self‐perceived learning outcomes, particularly self‐perceived knowledge and skills. Students' perceptions of what they have learned are important because they can influence their learning behaviour, engagement, confidence, and readiness to apply acquired knowledge and skills in clinical practice [7, 8]. Although self‐perceived competence is not the same as objectively measured competence, it provides valuable insight into how students understand their educational development and identify areas where further support may be needed [7, 8, 9]. Therefore, assessing radiography interns' and recent graduates' perceptions of their acquired knowledge and skills can help identify perceived strengths and areas for improvement in undergraduate radiography education.

Radiographers perform medical imaging examinations and contribute to the diagnostic process. Medical imaging plays a crucial role in diagnosing illnesses and developing treatment plans for patient care [3]. During clinical training, radiography interns rotate through hospital departments, including Computed Tomography (CT), Magnetic Resonance Imaging (MRI), X‐ray and ultrasound, where they gain clinical experience with different patient populations under the guidance of experienced radiographers [10]. In Saudi Arabia, radiography education began in 1976 in response to the need for graduates with a wide range of professional competencies [11].

Radiography interns require effective instruction, clinical training, and close supervision to support their transition into professional practice. Previous studies have reported challenges during radiography training, including inadequate training, limited supervisory support, and high workload [12, 13]. These challenges may affect the development of the knowledge and skills required for safe radiography practice and highlight the importance of evaluating interns' and recent graduates' perceptions of their professional competency development.

The professional domains assessed in this study include communication, procedural knowledge, technical knowledge, patient care, collaborative teamwork, medico‐legal and ethical knowledge, and identification of significant findings [4]. These domains are essential for safe and effective medical imaging practice. Understanding graduates' self‐perceived knowledge and skills may help universities identify perceived strengths and areas for improvement in undergraduate radiography education and better support graduate preparedness [14].

Therefore, this study aimed to investigate Saudi Radiological Sciences interns' and recent graduates' perceptions of their acquired knowledge and skills across seven professional domains: communication, procedural knowledge, technical knowledge, patient care, collaborative teamwork, medico‐legal and ethical knowledge, and identification of significant findings.

## 2 Methodology

### 2.1 Study Population

A cross‐sectional study was conducted in Saudi Arabia, among Radiological Sciences students at the College of Applied Medical Sciences across two Saudi universities (King Saud Bin Abdulaziz University for Health Sciences and King Abdulaziz University). The study was conducted over 1 year, beginning in September 2023, to allow sufficient time for recruitment and data collection. The sample size was determined by the accessible population of eligible radiography interns and recent graduates from the selected cohorts and institutions. Because this was a descriptive cross‐sectional study using convenience sampling, no formal power calculation was performed. All eligible participants from the 2020 to 2023 cohorts were invited to participate. Of the 150 eligible individuals, 61 completed the survey and were included in the final analysis, yielding a response rate of 40.7%. This sample was considered appropriate for the exploratory descriptive purpose of the study, which aimed to summarize participants' perceptions rather than test causal relationships or compare intervention effects.

Participation was limited to radiography students, specifically focusing on interns and recent graduates. Participants classified as radiography interns were those who had completed all academic requirements of a Radiological Sciences degree program and were actively engaged in a structured, supervised clinical internship as part of the requirements for graduation and professional registration. Recent graduates were defined as individuals who had successfully completed the Radiological Sciences program and belonged to the 2020–2023 graduation cohorts. The inclusion criteria were radiography interns and recent graduates from the 2020 to 2023 cohorts at the two selected Saudi universities who met the definitions provided above. Individuals who were not from radiography programs, had not reached the internship stage, or were outside the selected cohorts or institutions were not considered eligible for the study population. Participants who did not provide informed consent and responses with incomplete or missing data were excluded from the analysis. Recruitment was conducted through official email lists and WhatsApp groups associated with the Radiological Sciences programs at the two selected universities. The invitation message included a brief description of the study purpose, eligibility criteria, voluntary nature of participation, confidentiality statement, and a link to the online questionnaire, which was administered using Google Forms. The first page of the Google Form contained the participant information sheet and informed consent statement. Participants were required to confirm their consent before proceeding to the questionnaire. Eligibility was checked through screening questions asking participants to confirm their program affiliation, cohort year, and status as either a radiography intern or recent graduate. Only participants who met the inclusion criteria, provided consent, and completed the questionnaire were included in the final analysis.

Ethical approval for this study was obtained from the appropriate Institutional Review Board (approval number: SP23J‐122‐08). Participation was entirely voluntary, and informed consent was obtained electronically through the first page of the Google Form before participants proceeded to the questionnaire. Participants were informed of their right to withdraw from the study at any time without consequences. All responses were treated as anonymous and confidential.

### 2.2 Instrument

The primary construct measured in this study was interns' and recent graduates' perceptions of the knowledge and skills acquired during undergraduate radiography education. This construct was assessed across seven professional domains: radiographic procedural knowledge, radiographic technical knowledge, medico‐legal and ethical knowledge, communication skills, identification of significant findings, collaborative teamwork, and patient‐centred care. To provide a comprehensive evaluation of this primary construct, the questionnaire examined six related dimensions: perceived improvement, perceived importance, perceived inclusion within the degree program, perceived confidence, perceived contribution of clinical placement, and perceived future relevance. These dimensions were treated as subdomains reflecting participants' perceptions of acquired knowledge and skills, rather than as separate independent constructs.

The questionnaire and definitions of the seven professional competency domains were adapted from the Science Students Skills Inventory (SSSI), developed by Matthews and Hodgson, and later used by Pettit, Hodgson, and Williams [4, 9]. The SSSI is a previously validated instrument assessing students' perceptions of professional and transferable skills developed during undergraduate education. The questionnaire items were reworded and contextualized for radiological sciences while preserving the original conceptual domains. To ensure content relevance, clarity and face validity, the adapted questionnaire was reviewed by two radiologists and two senior radiology specialists with academic and clinical expertise. Minor wording refinements were made based on their feedback without altering the instrument domains. The questionnaire consisted of closed‐ended questions covering seven professional competency domains (Table 1).

**TABLE 1:** Definitions of the seven professional competency domains as they were presented in the survey.

Term	Definition
Radiographic procedural knowledge	Patient positioning, radiation protection, use of contrast media, and use of standard precautions
Radiographic technical knowledge	Knowledge of different modalities, exposure factors, and physics of medical imaging, applying ALARA principles
Medico‐legal and ethical knowledge	Keeping up to date with current medico‐legal issues, engaging in ethical decision making, and the duty of care to the patient
Communication	Communicating effectively with patients and staff, the ability to explain procedures and dangers of ionizing radiation, and building rapport with patients
Identification of significant findings	The ability to recognize anatomy and pathology and communicate significant or abnormal findings to the reporting doctor
Collaborative teamwork	Professional working relationships with other healthcare professionals recognize the appropriate time to seek assistance
Patient‐centred care	Identifies and acknowledges patients' needs, modifies procedures to accommodate patients, uses a respectful and empathetic approach, and demonstrates cultural awareness

The questions were:

1. Indicate your level of improvement in the following professional competency domains as a result of your degree?
2. How important is it to have the following professional competency domains as a result of your degree?
3. To what extent were the following professional competency domains included in your degree?
4. To what extent do you feel confident in the following professional competency domains as a result of the medical imaging degree?
5. To what extent do you feel clinical placement has contributed to developing the following professional competency domains?
6. To what extent do you think the following professional competency domains will be relevant in your future career?

For conceptual clarity, the assessed professional competencies were categorized into cognitive, psychomotor/clinical, and affective components. Cognitive components included radiographic procedural knowledge, radiographic technical knowledge, medico‐legal and ethical knowledge, and identification of significant findings. Psychomotor/clinical components reflected the application of knowledge and skills in patient‐centred care and clinical practice. Affective components included confidence, perceived importance, perceived future relevance, communication, and collaborative teamwork. Accordingly, the term ‘professional competencies’ was used to reflect the combined knowledge, skills and affective perceptions assessed in this study.

Responses to the six questionnaire items were recorded using a five‐point Likert scale ranging from strongly disagree to strongly agree.

### 2.3 Statistical Analysis

Data were analysed using JMP software. Descriptive statistics, including frequencies, percentages, Likert‐scale distributions, and mean ± standard deviation (SD), were used to summarize participants' demographic characteristics and perceptions across the seven professional competency domains. Mean scores were interpreted using the following five‐point Likert scale ranges: 1.00–1.80 = strongly disagree, 1.81–2.60 = disagree, 2.61–3.40 = neutral, 3.41–4.20 = agree, and 4.21–5.00 = strongly agree. Higher mean scores indicated more positive perceptions of the assessed professional competency domains.

## 3 Results

### 3.1 Characteristics of the Participants

A total of 61 interns and recent graduates participated in the study. Males comprised 54% (*n* = 33) of the sample, while females accounted for 46% (*n* = 28). All participants were aged between 21 and 26 years. Graduates represented the largest group of respondents at 56%, while interns made up 44%. Nearly half of the participants (44%) had GPAs between 4.50 and 5.00, with 8% opting not to disclose their GPA. The majority of participants (70%) were from King Saud bin Abdulaziz University for Health Sciences, whereas 30% were from King Abdulaziz University (see Table 2).

**TABLE 2:** Demographic variables of Radiological Sciences interns and recent graduates.

Measure	Item	(*n*) %
Gender	Female	(*n* = 28) 46%
	Male	(*n* = 33) 54%
Age (Years)	21 years	(*n* = 6) 7%
	22 years	(*n* = 17) 29%
	23 years	(*n* = 13) 22%
	24 years	(*n* = 13) 22%
	25 years	(*n* = 9) 15%
	26 years	(*n* = 3) 5%
Status	Intern	(*n* = 27) 44%
	2022 graduate	(*n* = 7) 11%
	2021 graduate	(*n* = 10) 16%
	2020 graduate	(*n* = 17) 28%
GPA	3.00–3.50	(*n* = 4) 7%
	3.51–4.00	(*n* = 11) 19%
	4.01–4.50	(*n* = 13) 22%
	4.50–5.00	(*n* = 26) 44%
	Prefer not to tell	(*n* = 5) 8%
University	King Abdulaziz University	(*n* = 18) 30%
	King Saud bin Abdulaziz University for Health Sciences	(*n* = 43) 70%

A five‐point Likert scale was used to assess participants' perceptions across six assessment dimensions and seven professional competency domains. Overall, participants reported generally positive perceptions across most domains, with mean scores mainly falling within the ‘agree’ or ‘strongly agree’ ranges (Table 3).

**TABLE 3:** Mean ± standard deviation (SD) scores for participants' perceptions across professional competency domains.

Assessment dimension	Communication	Procedural knowledge	Technical knowledge	Patient care	Collaborative teamwork	Medico‐legal and ethical knowledge	Identification of significant findings
Perceived improvement	4.31 ± 0.89	4.28 ± 0.92	4.31 ± 0.87	4.10 ± 1.09	4.11 ± 0.95	3.95 ± 1.04	3.84 ± 1.05
Perceived importance	4.36 ± 0.73	4.23 ± 0.90	4.46 ± 0.81	4.25 ± 0.88	4.38 ± 0.89	4.21 ± 0.86	4.43 ± 0.95
Program coverage/inclusion	4.18 ± 0.99	4.31 ± 0.87	4.18 ± 1.02	4.30 ± 0.84	4.21 ± 0.97	3.95 ± 1.04	3.87 ± 1.26
Confidence	4.18 ± 1.07	4.31 ± 0.87	4.34 ± 0.77	4.26 ± 0.90	4.21 ± 0.81	4.07 ± 0.91	3.87 ± 1.12
Clinical placement contribution	4.33 ± 0.85	4.26 ± 0.93	4.23 ± 0.90	4.38 ± 0.82	4.26 ± 0.98	4.21 ± 0.84	3.90 ± 1.09
Future relevance	4.44 ± 0.81	4.51 ± 0.89	4.16 ± 1.11	4.36 ± 0.93	4.38 ± 0.97	4.38 ± 0.80	4.36 ± 0.93

*Note:* Mean scores were interpreted using the following five‐point Likert scale ranges: 1.00–1.80 = strongly disagree, 1.81–2.60 = disagree, 2.61–3.40 = neutral, 3.41–4.20 = agree, and 4.21–5.00 = strongly agree. Higher mean scores indicate more positive perceptions of the assessed professional competency domains.

Perceived improvement was highest for communication and technical knowledge (mean ± SD: 4.31 ± 0.89 and 4.31 ± 0.87, respectively), followed by procedural knowledge (4.28 ± 0.92). Followed by procedural knowledge (4.28 ± 0.92). Collaborative teamwork (4.11 ± 0.95) and patient care (4.10 ± 1.09) also received positive ratings. In comparison, medico‐legal and ethical knowledge (3.95 ± 1.04) and identification of significant findings (3.84 ± 1.05) received comparatively lower mean scores, indicating relatively lower perceived improvement in these areas.

For perceived importance, all professional competency domains were rated positively, with mean scores ranging from 4.21 ± 0.86 to 4.46 ± 0.81. Technical knowledge received the highest importance rating (4.46 ± 0.81), followed by identification of significant findings (4.43 ± 0.95), collaborative teamwork (4.38 ± 0.89), and communication (4.36 ± 0.73). Medico‐legal and ethical knowledge received the lowest mean score in this dimension (4.21 ± 0.86), although it remained within the ‘strongly agree’ range.

Regarding program coverage or inclusion, the highest mean scores were reported for procedural knowledge (4.31 ± 0.87), patient care (4.30 ± 0.84), and collaborative teamwork (4.21 ± 0.97). Communication and technical knowledge both received favourable ratings (4.18 ± 0.99 and 4.18 ± 1.02, respectively). Medico‐legal and ethical knowledge (3.95 ± 1.04) and identification of significant findings (3.87 ± 1.26) received comparatively lower ratings, suggesting that these domains were perceived as less comprehensively covered within the undergraduate program.

Participants also reported generally positive confidence levels across the assessed professional competency domains. The highest confidence rating was observed for technical knowledge (4.34 ± 0.77), followed by procedural knowledge (4.31 ± 0.87), patient care (4.26 ± 0.90), and collaborative teamwork (4.21 ± 0.81). Communication (4.18 ± 1.07) and medico‐legal and ethical knowledge (4.07 ± 0.91) were also rated positively. Identification of significant findings recorded the lowest confidence score (3.87 ± 1.12), indicating comparatively lower perceived confidence in this domain.

For clinical placement contribution, patient care received the highest mean score (4.38 ± 0.82), followed by communication (4.33 ± 0.85), procedural knowledge (4.26 ± 0.93), collaborative teamwork (4.26 ± 0.98), technical knowledge (4.23 ± 0.90), and medico‐legal and ethical knowledge (4.21 ± 0.84). Identification of significant findings received the lowest score in this dimension (3.90 ± 1.09), although it remained within the ‘agree’ range.

For future relevance, procedural knowledge received the highest rating (4.51 ± 0.89), followed by communication (4.44 ± 0.81), collaborative teamwork (4.38 ± 0.97), medico‐legal and ethical knowledge (4.38 ± 0.80), patient care (4.36 ± 0.93), and identification of significant findings (4.36 ± 0.93). Technical knowledge received the lowest mean score in this dimension (4.16 ± 1.11), but it remained within the ‘agree’ range.

Overall, Table 3 shows that participants perceived most professional competency domains positively across all assessment dimensions. However, medico‐legal and ethical knowledge and identification of significant findings consistently received comparatively lower scores in perceived improvement, program coverage, confidence, and clinical placement contribution, highlighting areas that may benefit from further curricular and clinical enhancement.

## 4 Discussion

This study examined Saudi Radiological Sciences interns' and recent graduates' perceptions of professional competency development during their undergraduate education at two universities. Overall, participants reported positive perceptions across several professional competency domains, particularly communication, technical knowledge, procedural knowledge, collaborative teamwork, and patient care. These findings suggest that participants perceived these areas as relative strengths within their undergraduate education. However, because the findings are based on self‐reported perceptions, they should not be interpreted as objective evidence of program effectiveness, graduate competence, or clinical performance.

Medico‐legal and ethical knowledge and identification of significant findings received comparatively lower ratings in perceived improvement and program coverage. These findings indicate areas that may benefit from targeted curricular and clinical enhancement rather than demonstrating deficiencies in the program as a whole. Previous studies have also highlighted the importance of strengthening medico‐legal, ethical, and interpretive competencies in radiography education and clinical training [4, 15, 16]. Therefore, integrating focused learning activities related to medico‐legal and ethical principles during the internship phase may help reinforce professional awareness and support graduate preparedness.

Additionally, identification of significant findings was also one of the lowest‐scoring items concerning improvement, program coverage, confidence levels and clinical contribution. One possible explanation for this is that students may not have had enough workplace experience to gain confidence in this competency domain. Identifying significant findings is a higher‐order professional competency that requires radiographers to interpret and recognize urgent cases and effectively communicate these findings to the treating physician and radiologist so that the patient can receive prompt treatment. This finding is consistent with other studies indicating that students need further training in this competency domain [17, 18]. Although perceptions of this competency domain were still generally positive, the findings suggest that a targeted and comprehensive training component during the internship would be beneficial, particularly for students who feel less confident, to further strengthen competence and clinical readiness.

This study has several limitations. First, the relatively small sample size of 61 participants and the restriction of the sample to interns and recent graduates from two universities may limit the generalizability of the findings to the wider population of Saudi Radiological Sciences graduates. Second, participants were recruited from different cohorts between 2020 and 2023, which may have introduced cohort‐related variation, including potential differences in curriculum delivery, COVID‐19‐related disruption, and clinical placement exposure. These factors were not controlled using stratified or adjusted analysis because of the descriptive nature of the study and the limited sample size. Therefore, the findings should be interpreted as overall perception‐based results rather than evidence of differences between cohorts. Third, although the questionnaire was adapted from a previously validated instrument and reviewed by experts for content relevance and face validity, formal pilot testing and internal consistency reliability testing, such as Cronbach's alpha, were not conducted. Therefore, the psychometric properties of the adapted questionnaire were not fully established in this sample. Future studies with larger and more diverse samples should examine cohort‐specific differences, adjust for potential confounding factors, and further evaluate the reliability and validity of the adapted questionnaire in radiography student populations.

Despite these limitations, the findings of this research are encouraging in terms of improving graduates' knowledge and confidence in medico‐legal and ethical knowledge, as well as their ability to identify significant findings. Recommendations include reviewing the curriculum to emphasize these competency domains and developing appropriate teaching strategies. Integrating these competency domains into clinical placements may help address the weaknesses identified and increase students' understanding of how to practice them in the real world. Furthermore, providing faculty development opportunities would also be beneficial. These suggestions may help universities strengthen Radiological Sciences programs and better support graduate preparedness.

## 5 Conclusion

This study provides valuable perception‐based insight into radiography undergraduate education at two Saudi universities. Interns and recent graduates reported generally positive perceptions of professional competency development across most assessed domains, particularly communication, procedural knowledge, technical knowledge, patient care, and collaborative teamwork. These findings suggest that participants perceived their undergraduate education as supportive of their professional preparation. However, because the findings are based on self‐reported perceptions, they should not be interpreted as objective evidence of program effectiveness, graduate competence, clinical performance, or patient safety outcomes. Comparatively lower ratings for medico‐legal and ethical knowledge and identification of significant findings highlight important opportunities for further curricular and clinical enhancement. Overall, the findings may provide useful direction for strengthening graduate preparedness and supporting future curriculum review in radiography education.

## Funding

The authors have nothing to report.

## Ethics Statement

The Institutional Review Board (IRB) at King Abdullah International Medical Research Center has granted approval for this research under protocol number SP23J‐122‐08.

## Consent

Written informed consent is obtained from all participants prior to their completion of the questionnaire.

## Conflicts of Interest

The authors declare no conflicts of interest.

## Data Availability

The data that support the findings of this study are available from the corresponding author upon reasonable request.
